# The impact of clinical risk conditions on influenza and pneumonia diagnoses in England: a nationally representative retrospective cohort study, 2010–2019

**DOI:** 10.1017/S0950268822000838

**Published:** 2022-05-06

**Authors:** Darya Pokutnaya, Matthew M. Loiacono, Helen Booth, Rachael Williams, Christopher Ma, James Parker, Hélène Bricout, Susan Farrow, Joshua Nealon

**Affiliations:** 1 Department of Epidemiology, School of Public Health, University of Pittsburgh, Pittsburgh, PA, USA; 2 Global Medical Evidence Generation, Sanofi, Swiftwater, PA, USA; 3 Clinical Practice Research Datalink (CPRD), Medicines and Healthcare Products Regulatory Agency, London, UK; 4 UK/IE Medical Affairs, Sanofi, Reading, Berkshire, UK; 5 Global Medical Evidence Generation, Sanofi, Lyon, France; 6 School of Public Health, Li Ka Shing Faculty of Medicine, The University of Hong Kong, Hong Kong Special Administrative Region, China

**Keywords:** Co-morbidities, disease burden, influenza, pneumonia, retrospective cohort study

## Abstract

The impact of influenza and pneumonia on individuals in clinical risk groups in England has not previously been well characterized. Using nationally representative linked databases (Clinical Practice Research Database (CPRD), Hospital Episode Statistics (HES) and Office for National Statistics (ONS)), we conducted a retrospective cohort study among adults (≥ 18 years) during the 2010/2011–2019/2020 influenza seasons to estimate the incidence of influenza- and pneumonia-diagnosed medical events (general practitioner (GP) diagnoses, hospitalisations and deaths), stratified by age and risk conditions. The study population included a seasonal average of 7.2 million individuals; approximately 32% had ≥1 risk condition, 42% of whom received seasonal influenza vaccines. Medical event incidence rates increased with age, with ~1% of adults aged ≥75 years hospitalized for influenza/pneumonia annually. Among individuals with *vs.* without risk conditions, GP diagnoses occurred 2–5-fold more frequently and hospitalisations were 7–10-fold more common. Among those with obesity, respiratory, kidney or cardiovascular disorders, hospitalisation were 5–40-fold more common than in individuals with no risk conditions. Though these findings likely underestimate the full burden of influenza, they emphasize the concentration of disease burden in specific age and risk groups and support existing recommendations for influenza vaccination.

## Background

Influenza causes substantial burden in England during annual winter epidemics, especially amongst the elderly and those with pre-existing medical conditions, who are therefore prioritized for annual influenza vaccination [1–3]. Following recommendations from the Joint Committee on Vaccination and Immunization (JCVI), the National Health Service (NHS) offers free annual influenza vaccination to older adults (65+ years of age), people with clinical risk (herein ‘risk’) conditions (including pregnant women) irrespective of age, all children 2–10 years of age, those in long-stay residential care and carers or close contacts of immunocompromised individuals [4–6]. In response to the COVID-19 pandemic, free influenza vaccination was additionally offered to all adults aged 50–64 and to children aged 11 for the 2020/2021 season. This was then expanded to include children aged 12–15 for the 2021/2022 influenza season [7, 8].

Several prior studies have sought to describe and quantify influenza burden in risk groups in England using statistical modelling approaches [2, 9–11]. For example, Pitman *et al.* used regression techniques and estimated that 779 000–1 164 000 general practice (GP) visits, 19 000–31 200 hospitalisations and 18 500–24 800 deaths are attributable to influenza each year [11]. Cromer *et al.* used pathogen-specific attributable fractions to estimate a 2.7-fold increased risk of influenza-related hospitalisation among at-risk *vs*. non-at-risk individuals [2]. Adding to the complexity of characterizing influenza's impact, a proportion of disease attributable to influenza is diagnosed as pneumonia or other diseases, which contributes to underestimating its burden [12]. Viral influenza pneumonia is a rare complication of infection; secondary bacterial infection is far more common and has often been used as a sensitive case definition in influenza surveillance and burden studies, especially in studies quantifying deaths which suffer from particularly imprecise coding [13, 14]. No study to our knowledge has used a nationally representative database to directly describe influenza and pneumonia GP diagnoses, hospitalisations and deaths across the English population, stratified by age and specific risk conditions.

To improve understanding of populations at highest risk of influenza and its complications over recent years and to inform vaccination policies, we conducted a descriptive analysis of influenza- and pneumonia-diagnosed medical events in the adult population in England. We calculated the incidence rates (IRs) of GP diagnoses, hospitalisations and deaths across the population and estimated the additional risk contributed by the presence of risk conditions during influenza seasons 2010/2011–2019/2020.

## Methods

### Study design and data source

We conducted a retrospective cohort study using data extracted from the UK Clinical Practice Research Datalink (CPRD) GOLD and Aurum databases. CPRD GOLD and CPRD Aurum are longitudinal, nationally representative primary care databases that collected data from 1200 primary care practices covering approximately 23% of the English population at the time of this study (July 2020 database builds) [15]. The data were linked at the person-level to the Hospital Episodes Statistics Admitted Patient Care (HES APC) hospitalisation data and the Office for National Statistics (ONS) mortality data to provide information across the continuum of care [16]. The CPRD databases consist of pseudonymised patient electronic health records and therefore individual participant consent is not required [17]. Data were collected for 10 consecutive influenza seasons (defined as 1 December to 31 May of the following year) from 2010/2011 to 2019/2020. All adults (aged ≥18 years) registered with participating GP practices on 1 December of each season and eligible for linkage to HES APC and ONS databases were included in each seasonal analysis. HES APC and ONS data were not available for the 2019/2020 season. Further details of eligible patient selection are provided in Supplementary Figure S1.

### Study outcomes

The primary outcomes were influenza and pneumonia GP diagnoses, hospitalisations and deaths. Hospitalisations and deaths were captured with ICD-10-codes (influenza: J09–J11 and pneumonia: J12–J18). Primary care events were captured using SNOMED-CT codes which were mapped to the corresponding ICD-10 codes and verified by a trained clinical reviewer (CM) (SNOMED-CT codes available in Supplementary File 1). As some influenza SNOMED-CT codes are non-specific (i.e. ‘influenza or common cold’), influenza-like-illness GP diagnoses may also be captured. Any GP diagnoses coded in the individual's primary care record were captured, which may have occurred during or outside of a GP consultation, including information shared from secondary care visits. Only hospitalisations for ≥1 night were included. To increase the specificity of case ascertainment for hospitalisations, only primary discharge diagnoses (i.e. the main reason for hospitalisation) resulting in hospitalisation were included. Only the underlying cause of death was included. Events were stratified by age (18–34; 35–49; 50–64; 65–74; ≥ 75 years) and clinical risk conditions. Only first events occurring in each participant each season were captured, and the cohorts were re-defined at the beginning of every season. The total number of incident outcome events experienced by the study population was summed for each season.

### Definition of risk conditions

Risk conditions corresponded to eligibility for free influenza vaccination in England under the National Immunisation Programme. Included conditions were asthma, blood disorders (captured here as haemolytic anaemias or anaemia in chronic disease), cardiovascular disorders, diabetes, endocrine disorders, immunocompromised, kidney disorders, liver disorders, obesity, neurologic/neuromuscular disorders and respiratory conditions (ICD-10 codes or prescriptions used to define each risk condition available in Supplementary Table S1). Individuals were included in a risk group for a corresponding season if they were recorded as having a risk condition in the primary care data within 3 years of the start of the influenza season or had a diabetes prescription less than 6 months before the start of each season. Individuals could contribute to more than one risk group.

### Statistical methods

Descriptive statistics of study population characteristics were reported as seasonal averages, or the average number of events across seasons divided by the average number of total individuals across seasons. The seasonal average IRs for each outcome, expressed as rates per 100 000 population, and corresponding 95% confidence intervals (CIs) were estimated using Quasi-Poisson models. A Quasi-Poisson model was used to account for both the under- and over-dispersion we observed in the data following model fitting and subsequent testing procedures [18, 19]. Models were parameterized with the number of events as the dependent variable and no independent variables, thus estimating the model intercept only – the log seasonal total denominator population was specified as an offset. Incidence rate ratios (IRRs) and corresponding 95% CIs comparing rates in individuals with risk conditions *vs*. those with no risk conditions were similarly estimated using a Quasi-Poisson model. Due to a small number of deaths captured in the data, IRRs for deaths were not calculated. The CPRD co-authors had access to the databases to create the study population and measure the selected outcomes. Otherwise, the statistical analyses were performed using R Statistical Software (R version 4.0.2; RStudio version 1.3.1073; R Foundation for Statistical Computing, Vienna, Austria). The study protocol was approved by the Independent Scientific Advisory Committee (ISAC) at the Medicines and Healthcare products Regulatory Agency (Protocol #: 20_115 R0 A1).

## Results

### Study population characteristics

**Fig. 1.**
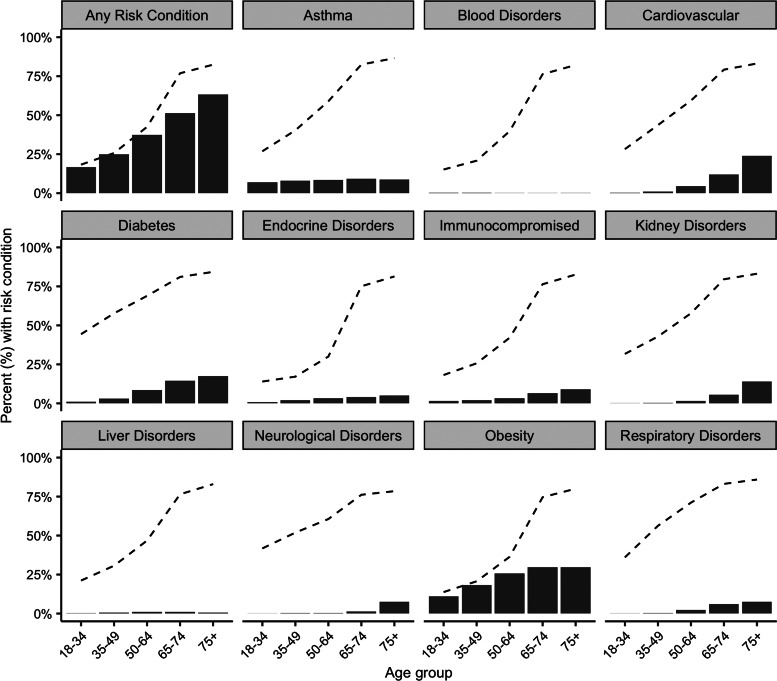
Seasonal average prevalence of risk conditions (solid bars) and influenza vaccination rates (dashed line), by age group, 2010/2011–2019/2020. Note: Underlying data provided in Supplementary Tables S2 and S4.

The study population included a seasonal average of 7 162 566 individuals over 18 years of age, 50.5% female (Supplementary Table S2). Overall, 32.1% (*n* = 2 297 078) of individuals had at least one risk condition, of which 49.2% (*n* = 1 131 075) had received a seasonal influenza vaccine. Prevalence of risk conditions increased with age, ranging from 16.7% among 18–34-year-olds to 63.3% among individuals ≥75 years of age (Fig. 1). The most common risk condition was obesity with an overall prevalence of 20.2%, followed by asthma (8.0%) and diabetes (6.4%). Vaccination rates among at-risk individuals were highest for those aged 65–74 (76.9%) and ≥75 years (82.4%), whereas rates notably declined for those aged <65 (ranging from 18.3% to 42.5%) years (Fig. 1; Supplementary Table S4). Among specific risk groups, vaccination rates were lowest for those with blood disorders (31.1%), obesity (41.6%) or endocrine disorders (44.2%), and were highest for those with neurological (74.4%), kidney (76.5%) or respiratory disorders (79.2%) (Supplementary Table S4).

### Overall counts and incidence rates of influenza and pneumonia

Across the 2010/2011–2019/2020 influenza seasons, there were a total of 28 494 influenza events (21 986 GP diagnoses, 6192 hospitalisations and 316 deaths) and 257 061 influenza/pneumonia (P&I) events (142 660 GP diagnoses, 105 054 hospitalisations and 9347 deaths) (Supplementary Table S3). Individuals in the 35–49 age group had the highest number of influenza GP diagnoses, while individuals aged ≥75 years had the highest number of influenza-related hospitalisations and deaths (Table 1). For P&I-related events, all occurred most frequently among individuals aged ≥75 years. Influenza GP diagnosis IRs increased with age from 24 per 100 000 (95% CI 15–37) in 18–34-year-olds to 57 (37–84) in individuals ≥75 years of age. Similarly, influenza hospitalisation IRs increased from 3 (1–6) to 34 (14–68) and death IRs increased from 0.0 (0.0–0.1) to 3.3 (1.3–6.5). Among 18–34-year-olds, ~44% of P&I GP diagnoses were for influenza. As age increased, the proportion of influenza within P&I decreased. Among individuals ≥75 years of age only ~6% of GP diagnoses for P&I were for influenza, a proportion which was similar for hospitalisations and deaths.

**Table 1.** Seasonal average number (#) of events and incidence rates (IR) of general practitioner (GP) diagnoses, hospitalisations and deaths per 100 000 population by age group, 2010/2011–2019/2020

Age group		18–34 yrs	35–49 yrs	50–64 yrs	65–74 yrs	≥ 75 yrs	All ages
GP diagnoses							
Influenza	#	507	542	500	250	400	2199
	IR	24 (15–37)	28 (20–39)	31 (22–42)	31 (21–43)	57 (37–84)	31 (21–43)
Pneumonia	#	634	1199	2084	2275	6044	12 236
	IR	30 (23–38)	63 (49–79)	127 (94–167)	282 (213–364)	862 (708–1038)	171 (111–250)
Pneumonia and influenza	#	1128	1720	2548	2492	6376	14 264
	IR	54 (40–70)	90 (71–112)	156 (118–201)	309 (233–399)	910 (742–1101)	199 (133–284)
Hospitalisations							
Influenza	#	67	90	151	139	241	688
	IR	3 (1–6)	5 (2–8)	9 (4–18)	17 (7–34)	34 (14–68)	10 (3–21)
Pneumonia	#	283	616	1473	2226	6431	11 029
	IR	13 (9–19)	32 (22–45)	90 (59–130)	275 (190–382)	914 (698–1171)	153 (85–249)
Pneumonia and influenza	#	348	702	1617	2356	6649	11 672
	IR	16 (11–24)	36 (24–52)	99 (64–144)	291 (199–407)	945 (717–1217)	162 (90–264)
Deaths							
Influenza	#	0	<5	<5	6	23	35
	IR	0.0 (0.0–0.1)	0.1 (0.0–0.3)	0.2 (0.1–0.4)	0.7 (0.2–1.5)	3.3 (1.3–6.5)	0.5 (0.1–1.2)
Pneumonia	#	<5	7	33	72	888	1002
	IR	0.1 (0.0–0.2)	0.4 (0.2–0.6)	2 (1.2–3.1)	8.9 (6–12.5)	126.2 (100–156.5)	13.9 (7.7–22.8)
Pneumonia and influenza	#	<5	10	37	78	911	1038
	IR	0.1 (0.0–0.2)	0.5 (0.3–0.8)	2.3 (1.4–3.5)	9.6 (6.4–13.8)	129.5 (102.2–161.2)	14.4 (7.9–23.7)

yrs, years. 95% confidence intervals (in parentheses) derived from Quasi-Poisson model. Hospitalisation and death data were only available for 2010/2011–2018/2019 influenza seasons.

To preserve patient confidentiality, cells containing less than five events are noted as ‘<5’ in the table above.

GP diagnosis, hospitalisation and death IRs varied by influenza season. Influenza GP diagnosis IRs, averaged across all age groups, ranged from 10.0 (8.6–11.6) per 100 000 in the 2013/2014 influenza season to 71.4 (62.1–81.4) in 2010/2011 (Table 2). Influenza hospitalisation IRs ranged from 0.6 (0.4–1.0) in the 2011/2012 influenza season to 32.6 (13.1–65.5) in 2017/2018. All influenza death IRs were below two for all seasons, peaking at 1.8 (0.4–5.1) in the 2017/2018 season. The highest incidence of P&I GP diagnoses, hospitalisations and deaths all occurred in 2018/2019.

**Table 2.** Seasonal incidence rates for general practitioner (GP) diagnoses, hospitalisations and deaths per 100 000 population, averaged across all age groups, 2010/2011–2019/2020

	Influenza			Pneumonia and influenza			Predominant circulating strain^a^
Season	GP diagnoses	Hospitalisation	Deaths	GP diagnoses	Hospitalisation	Deaths	
2010/11	71.4 (62.1–81.4)	6.1 (3.9–9.0)	0.2 (0.1–0.3)	197.8 (123.4–297.4)	104.1 (41.4–201.9)	9.8 (2.2–26.5)	A (H1N1)
2011/12	18.6 (14.7–23.1)	0.6 (0.4–1.0)	0.0 (0.0–0.1)	151.6 (71.4–277.0)	115.7 (38.3–259.4)	12.4 (2.4–36.0)	A (H3N2)
2012/13	29.4 (24.0–25.6)	1.4 (0.9–2.2)	0.1 (0.0–0.2)	171.2 (82.6–307.6)	134.5 (43.8–304.4)	14.0 (2.5–42.0)	A (H1N1)
2013/14	10.0 (8.6–11.6)	1.2 (0.7–1.8)	0.1 (0.0–0.2)	140.1 (63.2–262.9)	131.7 (43.1–297.5)	11.7 (2.3–34.0)	A (H1N1)
2014/15	16.5 (12.8–20.9)	4.1 (1.9–7.5)	0.4 (0.1–1.0)	184.4 (82.5–347.5)	175.4 (56.1–401.0)	18.5 (3.5–54.6)	A (H3N2)
2015/16	17.8 (14.8–21.2)	6.8 (4.0–10.5)	0.4 (0.2–0.8)	192.2 (92.3–346.4)	184.3 (64.1–402.8)	15.3 (3.1–43.2)	A (H1N1)
2016/17	16.0 (9.5–24.8)	7.4 (3.0–14.8)	0.4 (0.1–1.1)	204.4 (88.0–394.6)	191.0 (61.1–436.4)	16.1 (2.9–47.9)	A (H3N2)
2017/18	54.4 (33.4–82.6)	32.6 (13.1–65.5)	1.8 (0.4–5.1)	272.5 (121.9–513.7)	219.6 (71.6–496.9)	18.8 (3.7–54.5)	B and A (H3N2)
2018/19	39.5 (26.3–56.6)	26.2 (13.2–46.0)	0.9 (0.2–2.5)	240.9 (110.1–448.2)	205.5 (70.9–450.8)	13.3 (2.6–38.3)	A (H1N1)
2019/20	32.1 (20.4–47.6)	NA	NA	241.6 (110.4–449.7)	NA	NA	A (H3N2)

95% confidence intervals (in parentheses) derived from Quasi-Poisson model. Hospitalisation and death data were only available for 2010/2011–2018/2019 influenza seasons.

a Data on predominant circulating strains were sourced from: Public Health England, Annual flu reports (2021) (available at https://www.gov.uk/government/statistics/annual-flu-reports).

### Incidence rate ratios in risk conditions *vs*. no risk conditions

**Fig. 2.**
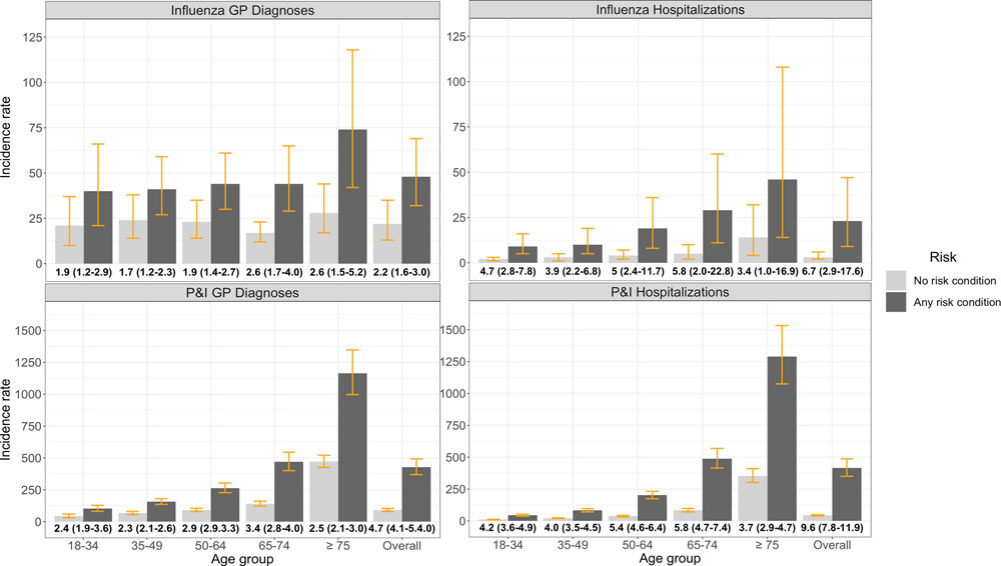
Seasonal average influenza and ‘pneumonia & influenza’ (P&I) general practitioner (GP) diagnosis and hospitalisation incidence rates per 100 000 population and incidence rate ratios (bolded values below *x*-axis, with 95% CIs), stratified by risk status and age group. Note: Yellow lines represent incidence rate 95% confidence intervals (CIs).

Individuals with any risk condition had consistently higher influenza and P&I IRs across all age groups compared to individuals with no risk conditions (Fig. 2). Influenza GP diagnosis IRRs comparing rates in those with risk conditions *vs*. those without ranged from 1.9 (1.2–2.9) among 18–34-year-olds to 2.6 (1.5–5.2) among individuals ≥75 years of age. Influenza hospitalisation IRRs were highest among individuals 65–74 years of age (5.8, 2.0–22.8) and 50–64 years of age (5.0, 2.4–11.7). The increased IR of P&I GP diagnoses and hospitalisation was also highest among 65–74-year-olds (3.4, 2.8–4; and 5.8, 4.7–7.4, respectively) and 50–64-year-olds (2.9, 2.9–3.3; and 5.4, 4.6–6.4).

**Fig. 3.**
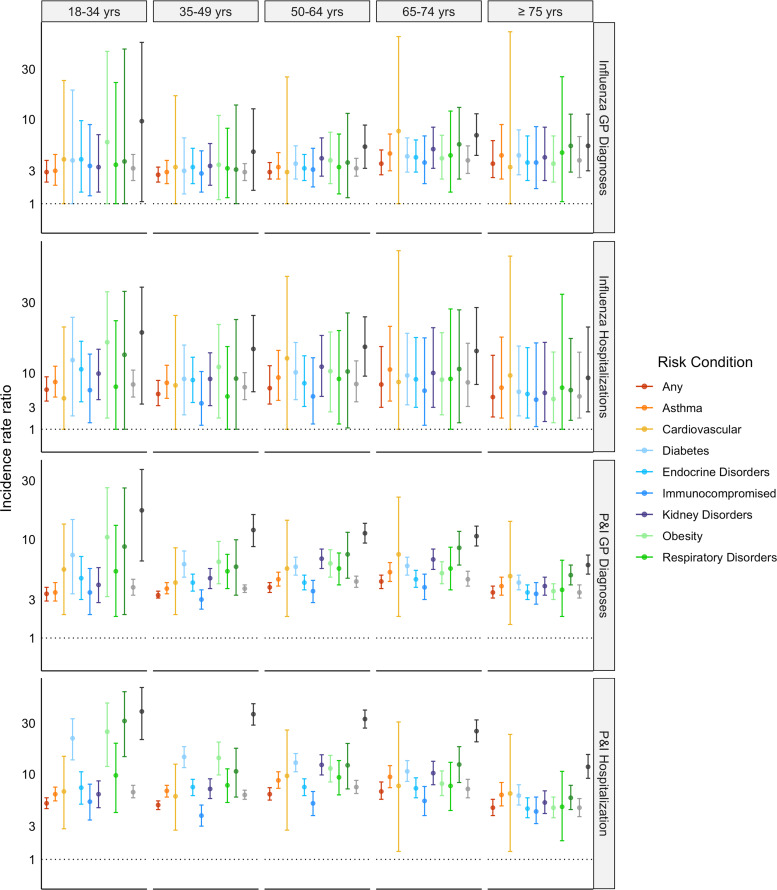
Influenza and ‘pneumonia & influenza’ (P&I) incidence rate ratios for general practitioner (GP) diagnoses and hospitalisations by specific risk condition *vs*. no risk condition (reference group), stratified by age group. yrs, years; any, any risk condition. Blood disorders, liver disorders and neurological disorders were excluded because they occur in ~<1% of the study population. *Y*-axis scale was reduced to improve readability. The full figure is available in the Supplementary Figure S2.

Influenza GP diagnosis and hospitalisation IRRs for individual risk conditions were >1 across all age groups (Fig. 3). Influenza hospitalisation IRRs were ~2-fold larger than GP diagnosis IRRs, with a similar age pattern. The P&I GP diagnosis and hospitalisations IRRs followed similar trends to influenza IRRs but were ~1–4-fold larger. Across all categories, the IRRs for asthma and immunocompromised individuals increased with age whereas the IRRs for cardiovascular, kidney and respiratory disorders decreased with age.

## Discussion

Using nationally representative databases, we described influenza- and pneumonia- diagnosed medical events in the adult population in England across the 2010/2011–2019/2020 influenza seasons, stratified by clinical risk groups and age. We observed an increase in the incidence of events with age, with the highest rates in adults ≥75 years old and in people with one or more risk conditions, reinforcing the importance of the existing policy recommendations to vaccinate these groups.

Exploring the interaction between age and the presence of risk conditions revealed additional nuances to these relationships. For example, the impact of a risk condition was higher for adults in the 50–64- and 65–74-year-old age groups than in those ≥75 years of age. This is possibly because of higher underlying rates of disease in the oldest adults – who may suffer from frailty or other risk factors for medically attended disease – irrespective of the presence of the risk conditions identified in our study. While influenza vaccination rates among the at-risk 65–74-year-olds (76.9%) and ≥75 age group (82.9%) were aligned with WHO targets to vaccinate 75% of those at highest risk [20], fewer than half of at-risk 50–64-year-olds were vaccinated each year. Approximately 35% of 50–64-year-olds had at least one risk condition, the presence of which was associated with ~5-fold elevated hospitalisation risk in this age group.

We further explored these relationships via stratification by specific risk conditions and identified elevated rates of clinical events which were not accompanied by correspondingly high vaccine coverage. Adults aged 18–34 years with underlying respiratory, kidney or cardiovascular disease, for example, had up to 35-fold higher diagnosis rates than individuals without risk conditions, but fewer than 40% had received a seasonal influenza vaccine. Perhaps GPs perceive these younger adults at lower risk of infection than older individuals and deprioritize them for influenza vaccination; or influenza vaccination may be outside the scope of care delivered by clinical specialists with whom these individuals are in typical contact [21].

We chose not to compare outcome rates between vaccinated and unvaccinated individuals because our study was not designed to calculate vaccine effectiveness (VE). Any such comparison would be liable to strong confounding by indication and ‘healthy vaccinee’ biases by which the baseline health characteristics of vaccine recipients – rather than their vaccination status – predict their health outcomes [22]. Indeed, we previously observed up to fourfold elevated rates of cardiovascular and up to ninefold higher rates of respiratory disease in younger adult influenza vaccine recipients than non-recipients, presumably due to English recommendations for influenza vaccine only in adults with existing chronic health conditions who are relatively likely to suffer hospitalisations [23]. In contrast, randomized experiments, free of these biases, measuring the efficacy of influenza vaccination on preventing cardiovascular events have found strong protective effects deserving of future research [24, 25]. Our results further highlight the need for efforts to improve influenza vaccination uptake in younger, at-risk individuals who are currently under-vaccinated, as to reduce the frequency of influenza, pneumonia and related hospitalisations and support healthcare system decongestion.

Over the duration of study period, we observed substantial variation in GP diagnoses and hospitalisation rates, exhibiting patterns which were unexpected. For example, the highest rates of GP diagnoses were recorded in the 2010/2011 season, yet the highest rates of hospitalisation were in 2017/2018. Influenza hospitalisation rates remained relatively constant from the 2012/2013 to 2013/2014 season, yet the rate of GP diagnoses decreased nearly threefold. Influenza severity is determined by vaccination and infection history, their relationship with antigenically similar/distinct viruses and host and viral genetics [26]. The 2017/2018 season was characterized by co-circulation of A(H3N2) and B viruses. Elevated hospitalisation rates may be in part explained by A (H3N2) infection leading to more severe disease in older adults, and a lower than anticipated influenza VE during this season, as a result of mismatch between the vaccine and circulating viral strains [27].

Our study has several limitations. While we used a robust set of codes to identify risk conditions, their prevalence may be over- or under-estimated due to how these conditions are captured in the data *vs*. the definitions of high-risk conditions used by GPs. For example, in England GPs are incentivized to maintain a register of obese patients, although we know this is an underestimate of population rates [28]. Additionally, BMI is usually recorded at GP registration or during NHS health checks for older adults but may be absent or outdated for individuals who do not attend these routine medical appointments. We used a broad set of ‘obesity’ SNOMED codes (see Supplementary File 1) combined with BMI recording to increase sensitivity, but it is possible that obesity as captured in our study may not match the eligibility criteria applied in practice for free influenza vaccination in England. Additionally, we defined influenza seasons slightly differently from the standard surveillance definition, for operational simplicity. Our seasonal definitions were slightly later and captured all epidemics during the study period, so we do not anticipate this design choice materially affected the results. It is also possible that some vaccines administered outside of the GP setting may not have been captured, but in comparison with influenza vaccine coverage rates reported by Public Health England, we anticipate only a small degree of underestimation [29].

The IRs reported here are likely underestimates of the full burden of influenza. For GP influenza diagnoses, we only captured those which were specific to ‘influenza’. However, as influenza confirmatory testing is not routinely conducted in primary care in the UK, we expect many respiratory episodes which may have been caused by influenza will have been recorded using non-specific respiratory codes [30]. This lack of specific laboratory confirmation is a limitation inherent to retrospective studies. Large, prospective surveillance studies would be a worthy avenue of future research to better understand influenza infection, milder disease and transmission dynamics more completely in England, as was recently done in South Africa, providing a granular view of household transmission frequency [31].

For hospitalisations, we only captured the primary discharge diagnosis which will also lead to under-reporting because admissions for which influenza is a contributor, but not the main cause of hospitalisation, will not have been captured in our study. Findings from prior excess modelling research illustrate the degree to which hospitalisations may have been underestimated. For example, Matias *et al.* estimated seasonal average respiratory burden hospitalisation rates in the UK attributable to influenza A (42 per 100 000) and B (2 per 100 000) [32]. Influenza hospitalisation rates observed in our study, where all seasons were characterized by predominant circulation of influenza A strains, peaked at 32.6 per 100 000 in 2017/2018, and in seven of the included seasons were <8 per 100 000, ~5–50-fold lower than rates presented by Matias *et al*. [32].

Similarly, many deaths due to influenza-related complications often go unreported, as physicians are not required to wait for laboratory-confirmation of influenza infection prior to completing the death certificate and influenza-related complications may occur weeks or months after the initial infection [33, 34]. As evidence of the scale of underestimation, prior excess modelling research has estimated global seasonal influenza-associated respiratory mortality rates ranging from 4.0 to 8.8 per 100 000, whereas rates observed in our study were several orders of magnitude lower, ranging from 0.0 to 1.8 [35].

The findings of this study likely only characterize a portion of the respiratory burden of influenza and did not attempt to quantify extra-pulmonary burden [36]. Nonetheless, our findings provide a complete overview of the incidence of influenza- and pneumonia-diagnosed medical events in England, emphasizing the concentration of disease burden in specific age and risk groups.

## Data Availability

These data were obtained from national EHR sources which are subject to local laws and regulations. UK data were provided by Clinical Practice Research Datalink (CPRD) under a license from the UK Medicines and Healthcare products Regulatory Agency. CPRD data can be obtained by researchers following a successful application to CPRD.

## Supplementary material

For supplementary material accompanying this paper visit http://doi.org/10.1017/S0950268822000838. click here to view supplementary material
