# Supplementary material for: The impact of clinical risk conditions on influenza and pneumonia diagnoses in England: a nationally representative retrospective cohort study, 2010–2019
Source: Epidemiol Infect. 2022 May 6;150:e107. doi: 10.1017/S0950268822000838 (PMC9171903; doi:10.1017/S0950268822000838)
Supplement: Supplementary file 1 [file S0950268822000838sup001.docx]

**Title: The impact of clinical risk conditions on influenza and pneumonia diagnoses in England: a nationally-representative retrospective cohort study, 2010 – 2019**

**Supplementary Information**

**Supplementary File 1.** *SNOMED-CT code list used to identify risk conditions and outcomes in primary care setting.*

**Supplementary Table 1.** *ICD-10 codes and prescriptions applied to define risk conditions.*

| **Risk condition** | **ICD-10 codes or prescriptions** |
| --- | --- |
| **Definition:** Codes recorded as UK primary care database within 3 years of the start of each influenza season. | |
| ***Asthma*** | J45 - Asthma |
| ***Blood disorders*** | D55-D59 - Hemolytic anemias D63 - Anemia in chronic disease |
| ***Cardiovascular*** | I20-I25 - Ischemic heart disease I50 - Heart failure I42-I43 - Cardiomyopathy I34-I37 - Valvular disease I48 - Atrial fibrillation I60-I69 - Cerebrovascular disease I70, I74 - Peripheral vascular disease I27-I28 - Pulmonary circulation disease Q251 - Coarctation of the aorta Q201 - Double outlet right ventricle  Q203 - D-transposition  Q225 - Ebstein anomaly Q234 - Hypoplastic left heart Q234 - Hypoplastic left heart Q205 - Single ventricle Q262 - TAPV Q231 - Tetralogy of fallot Q224 - Tetralogy of fallot Q200 - Truncus arteriosus |
| ***Diabetes*** | E10-E14  Prescription for glucose lowering medication (ATC A10) 6 months prior to inclusion (season start) |
| ***Endocrine disorders*** | E03 - Other hypothyroidism E04 - Other nontoxic goitre E05 - Thyrotoxicosis [hyperthyroidism] E06 - Thyroiditis E27 - Other disorders of adrenal gland |
| ***Immunocompromised*** | B20-B24 - HIV C00-C97 - Cancer L93 - Lupus M05-M06 - RA  Z940-Z944, Z948A - Solid organ transplant  K50-K51 - inflammatory bowel disease |
| ***Kidney*** | N18-N19 - chronic kidney disease Z49 - Care involving dialysis Z99.2 - Dependence on renal dialysis I12 - Hypertensive renal disease |
| ***Liver disorders*** | K70 - Alcoholic liver disease K713, K714, K715, K716, K717 - Chronic toxic liver conditions K721, K729 - Chronic hep failure K73 - Chronic hepatitis K74 - Cirrhosis K766, K769 - Portal hypertension B18-B19 - Chronic and unspecified viral hepatitis |
| ***Neurological/neuromuscular*** | G20 - Parkinsons disease G35 - MS G710 - Muscular dystrophy G30, F00 - Alzheimers F01-F02 - Dementia G122 - ALS |
| ***Obesity*** | E66 – Overweight and obesity |
| ***Respiratory*** | J43 - Emphysema J44 - COPD) J47 - Bronchiectasis E84 - Cystic fibrosis J81 - Pulmonary oedema J84 - Interstitial lung disease J85 - Abscess of lung and mediastinum A15, A16 - Respiratory tuberculosis Z94.2 - Lung transplant status  D86.0 - Sarcoidosis of lung |

**Supplementary Table 2.** *Seasonal average study population (#) and percentage (%) by age group and risk condition, 2010/11-2019/20.*

| Age group: |  | 18-34 yrs | 35-49 yrs | 50-64 yrs | 65-74 yrs | ≥ 75 yrs | All ages |
| --- | --- | --- | --- | --- | --- | --- | --- |
| Study population | # | 2,104,794 | 1,911,551 | 1,637,739 | 807,737 | 700,745 | 7,162,566 |
| Sex (female) | % | 49.9 | 48.8 | 49.3 | 51.5 | 58.3 | 50.5 |
| No risk condition | # | 1,753,958 | 1,434,895 | 1,026,526 | 393,239 | 256,869 | 4,865,488 |
|  | % | 83.3 | 75.1 | 62.7 | 48.7 | 36.7 | 67.9 |
| Any risk condition | # | 350,836 | 476,656 | 611,213 | 414,498 | 443,876 | 2,297,078 |
|  | % | 16.7 | 24.9 | 37.3 | 51.3 | 63.3 | 32.1 |
| Asthma | # | 147,837 | 150,414 | 138,785 | 73,624 | 61,819 | 572,479 |
|  | % | 7.0 | 7.9 | 8.5 | 9.1 | 8.8 | 8.0 |
| Blood disorders | # | 3,526 | 2,907 | 1,615 | 705 | 791 | 9,544 |
|  | % | 0.2 | 0.2 | 0.1 | 0.1 | 0.1 | 0.1 |
| Cardiovascular | # | 4,879 | 18,270 | 74,982 | 97,084 | 168,112 | 363,329 |
|  | % | 0.2 | 1.0 | 4.6 | 12.0 | 24.0 | 5.1 |
| Diabetes | # | 19,644 | 57,059 | 138,877 | 117,381 | 122,763 | 455,724 |
|  | % | 0.9 | 3.0 | 8.5 | 14.5 | 17.5 | 6.4 |
| Endocrine disorders | # | 18,606 | 38,413 | 52,182 | 33,160 | 35,617 | 177,979 |
|  | % | 0.9 | 2.0 | 3.2 | 4.1 | 5.1 | 2.5 |
| Immunocompromised | # | 32,225 | 37,256 | 54,814 | 53,623 | 63,222 | 241,140 |
|  | % | 1.5 | 1.9 | 3.3 | 6.6 | 9.0 | 3.4 |
| Kidney disorders | # | 1,725 | 6,806 | 25,590 | 45,109 | 98,575 | 177,805 |
|  | % | 0.1 | 0.4 | 1.6 | 5.6 | 14.1 | 2.5 |
| Liver disorders | # | 3,474 | 11,211 | 17,786 | 8,275 | 3,594 | 44,340 |
|  | % | 0.2 | 0.6 | 1.1 | 1.0 | 0.5 | 0.6 |
| Neurological disorders | # | 1,341 | 3,902 | 7,310 | 11,362 | 52,028 | 75,943 |
|  | % | 0.1 | 0.2 | 0.4 | 1.4 | 7.4 | 1.1 |
| Obesity | # | 233,119 | 346,649 | 420,477 | 240,025 | 207,690 | 1,447,960 |
|  | % | 11.1 | 18.1 | 25.7 | 29.7 | 29.6 | 20.2 |
| Respiratory disorders | # | 1,673 | 7,570 | 37,844 | 48,782 | 53,260 | 149,128 |
|  | % | 0.1 | 0.4 | 2.3 | 6.0 | 7.6 | 2.1 |

### **Supplementary Table 3.** *Total number of influenza and ‘pneumonia & influenza’ events recorded for the 2010/11 – 2019/20 influenza seasons, stratified by age group and no vs any risk condition.*

| Age group: | 18-34 yrs | 35-49 yrs | 50-64 yrs | 65-74 yrs | ≥ 75 yrs | All ages |
| --- | --- | --- | --- | --- | --- | --- |
| Influenza |  |  |  |  |  |  |
| GP diagnoses |  |  |  |  |  |  |
| No risk condition | 3,677 | 3,459 | 2,330 | 665 | 718 | 10,849 |
| Any risk condition | 1,394 | 1,962 | 2,671 | 1,830 | 3,280 | 11,137 |
| Hospitalizations |  |  |  |  |  |  |
| No risk condition | 309 | 353 | 342 | 178 | 322 | 1,504 |
| Any risk condition | 290 | 453 | 1,019 | 1,076 | 1,850 | 4,688 |
| Deaths |  |  |  |  |  |  |
| No risk condition | 0 | 8 | 9 | <5 | 24 | 43 |
| Any risk condition | <5 | 12 | 27 | 49 | 183 | 273 |
| Pneumonia & Influenza |  |  |  |  |  |  |
| GP diagnoses |  |  |  |  |  |  |
| No risk condition | 7,657 | 9,730 | 9,394 | 5,498 | 12,121 | 44,400 |
| Any risk condition | 3,617 | 7,474 | 16,094 | 19,426 | 51,649 | 98,260 |
| Hospitalizations |  |  |  |  |  |  |
| No risk condition | 1,703 | 2,729 | 3,461 | 2,983 | 8,244 | 19,120 |
| Any risk condition | 1,427 | 3,591 | 11,092 | 18,223 | 51,601 | 85,934 |
| Deaths |  |  |  |  |  |  |
| No risk condition | 10 | 31 | 66 | 98 | 1,361 | 1,566 |
| Any risk condition | 11 | 56 | 271 | 605 | 6,838 | 7,781 |

### **Supplementary Table 4.** *Seasonal average study population (#) and percentage (%) vaccinated by age group, with/without risk conditions, 2010/11 to 2019/20.*

| Age group: |  | 18-34 yrs | 35-49 yrs | 50-64 yrs | 65-74 yrs | ≥ 75 yrs | All ages |
| --- | --- | --- | --- | --- | --- | --- | --- |
| Study population | # | 2,104,794 | 1,911,551 | 1,637,739 | 807,737 | 700,745 | 7,162,566 |
| Total vaccinated | # | 108,150 | 167,567 | 336,356 | 553,404 | 549,528 | 1,715,005 |
|  | % | 5.1 | 8.8 | 20.5 | 68.5 | 78.4 | 23.9 |
| No risk condition | # | 43,970 | 44,931 | 76,476 | 234,730 | 183,823 | 583,930 |
|  | % | 2.5 | 3.1 | 7.4 | 59.7 | 71.6 | 12.0 |
| Any risk condition | # | 64,181 | 122,635 | 259,880 | 318,674 | 365,705 | 1,131,075 |
|  | % | 18.3 | 25.7 | 42.5 | 76.9 | 82.4 | 49.2 |
| Asthma | # | 39,730 | 60,619 | 81,802 | 60,714 | 53,517 | 296,382 |
|  | % | 26.9 | 40.3 | 58.9 | 82.5 | 86.6 | 51.8 |
| Blood disorders | # | 535 | 605 | 642 | 538 | 650 | 2,970 |
|  | % | 15.2 | 20.8 | 39.8 | 76.3 | 82.2 | 31.1 |
| Cardiovascular | # | 1,378 | 7,972 | 44,489 | 76,872 | 139,768 | 270,479 |
|  | % | 28.2 | 43.6 | 59.3 | 79.2 | 83.1 | 74.4 |
| Diabetes | # | 8,717 | 32,948 | 95,726 | 95,150 | 103,502 | 336,043 |
|  | % | 44.4 | 57.7 | 68.9 | 81.1 | 84.3 | 73.7 |
| Endocrine disorders | # | 2,601 | 6,579 | 15,659 | 24,899 | 28,978 | 78,716 |
|  | % | 14.0 | 17.1 | 30.0 | 75.1 | 81.4 | 44.2 |
| Immunocompromised | # | 5,856 | 9,568 | 23,054 | 40,963 | 52,209 | 131,650 |
|  | % | 18.2 | 25.7 | 42.1 | 76.4 | 82.6 | 54.6 |
| Kidney disorders | # | 547 | 2,916 | 14,774 | 35,893 | 81,914 | 136,044 |
|  | % | 31.7 | 42.8 | 57.7 | 79.6 | 83.1 | 76.5 |
| Liver disorders | # | 735 | 3,439 | 8,327 | 6,330 | 2,982 | 21,813 |
|  | % | 21.2 | 30.7 | 46.8 | 76.5 | 83.0 | 49.2 |
| Neurological disorders | # | 560 | 2,025 | 4,430 | 8,654 | 40,822 | 56,491 |
|  | % | 41.8 | 51.9 | 60.6 | 76.2 | 78.5 | 74.4 |
| Obesity | # | 32,092 | 71,682 | 152,952 | 179,090 | 166,231 | 602,047 |
|  | % | 13.8 | 20.7 | 36.4 | 74.6 | 80.0 | 41.6 |
| Respiratory disorders | # | 603 | 4,258 | 26,911 | 40,559 | 45,764 | 118,095 |
|  | % | 36.0 | 56.2 | 71.1 | 83.1 | 85.9 | 79.2 |

### **Supplementary Figure 1.** *Flow chart of eligible patient identification in Aurum and CPRD GOLD databases.*

*
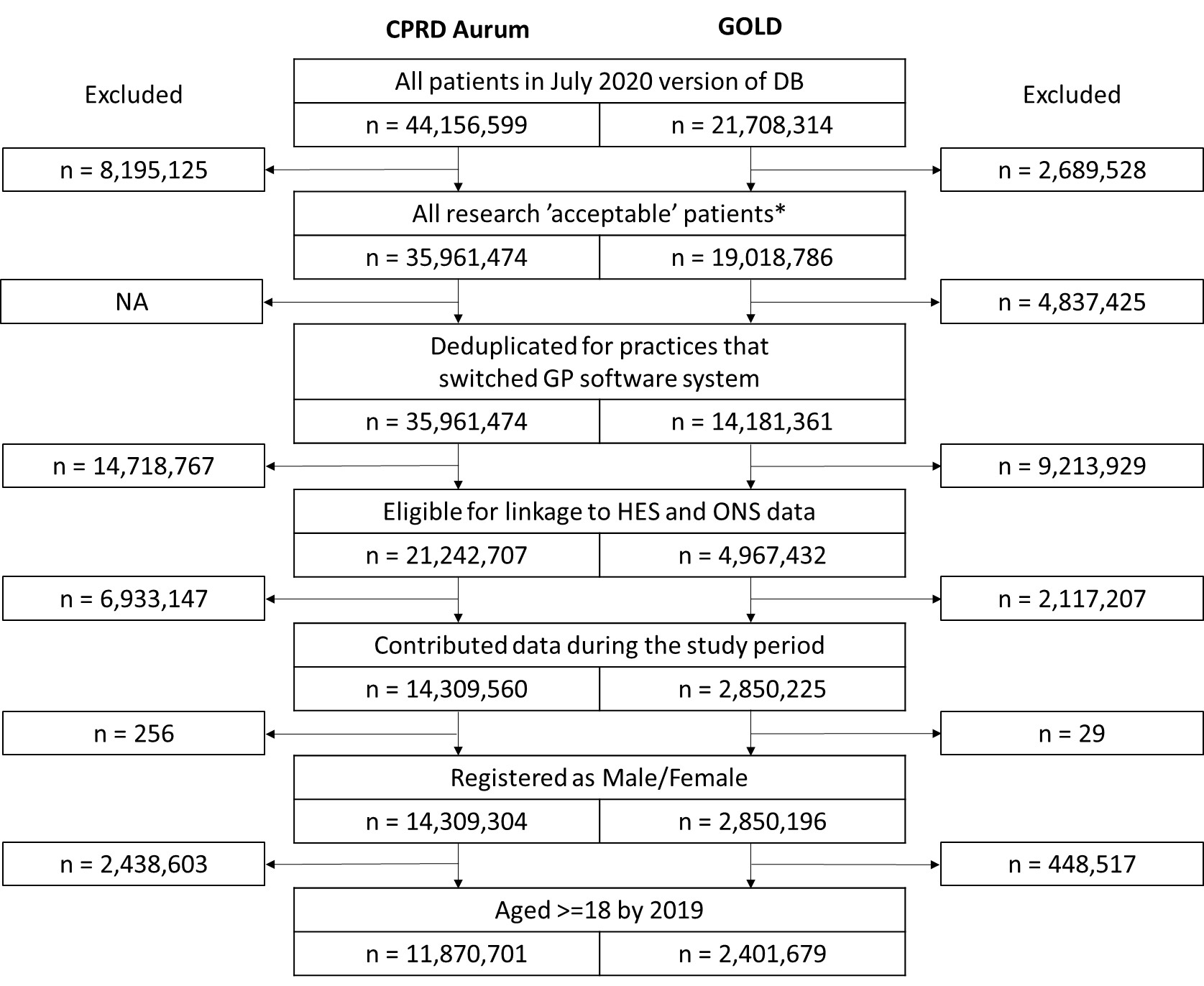
*

*Abbreviations: CPRD = Clinical Practice Research Database; DB = database; GP = general practitioner; HES = Hospital Episode Statistics; NA = not applicable, ONS = Office of National Statistics.*

******* *“Acceptable” patient for research use, as determined by CPRD*

**Supplemental Figure 2.** *Pneumonia and influenza (P&I) incidence rate ratios (log scale) for general practitioner (GP) diagnoses and hospitalizations by specific risk condition vs no risk condition (reference group), stratified by age group.*


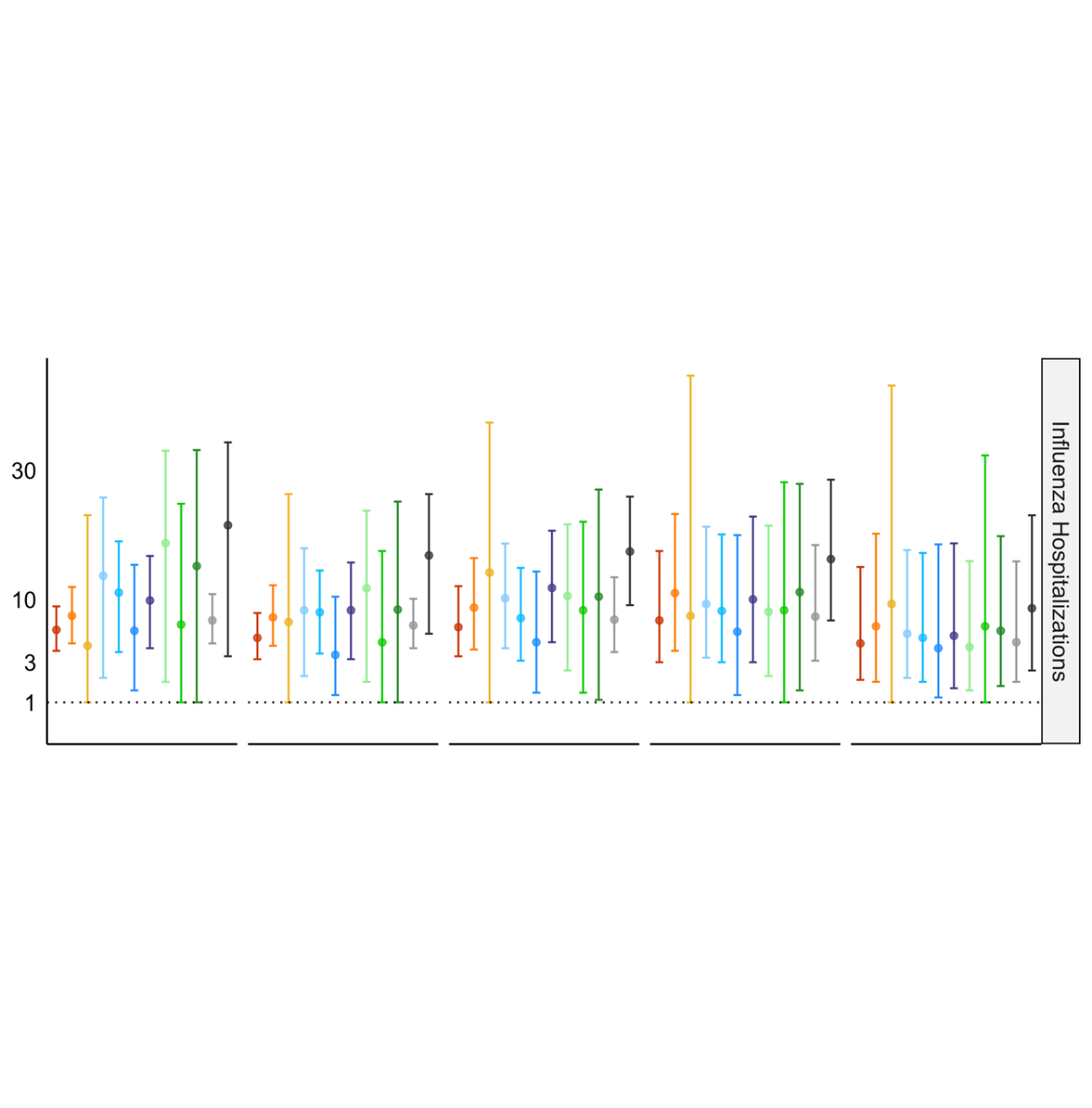

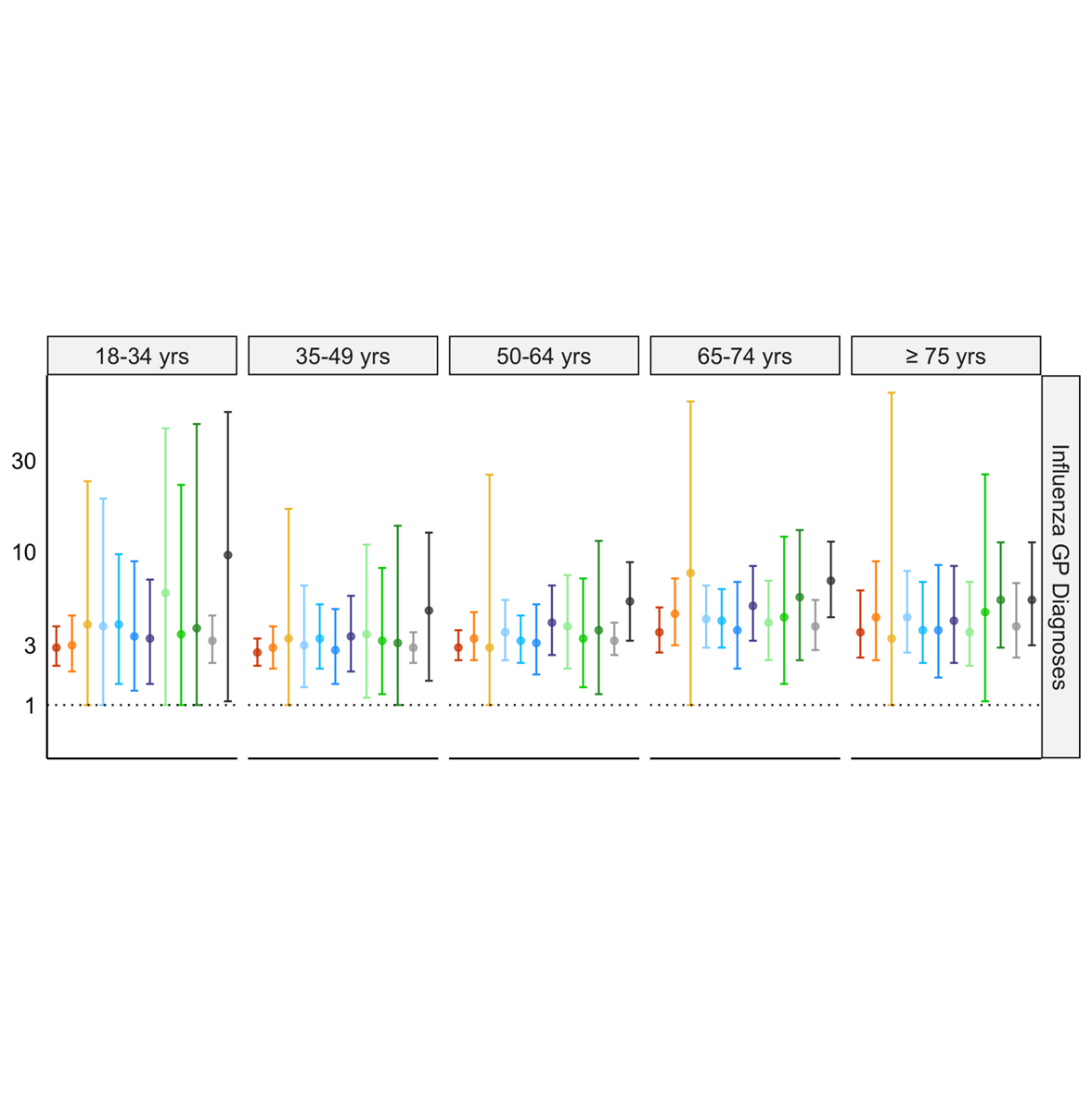

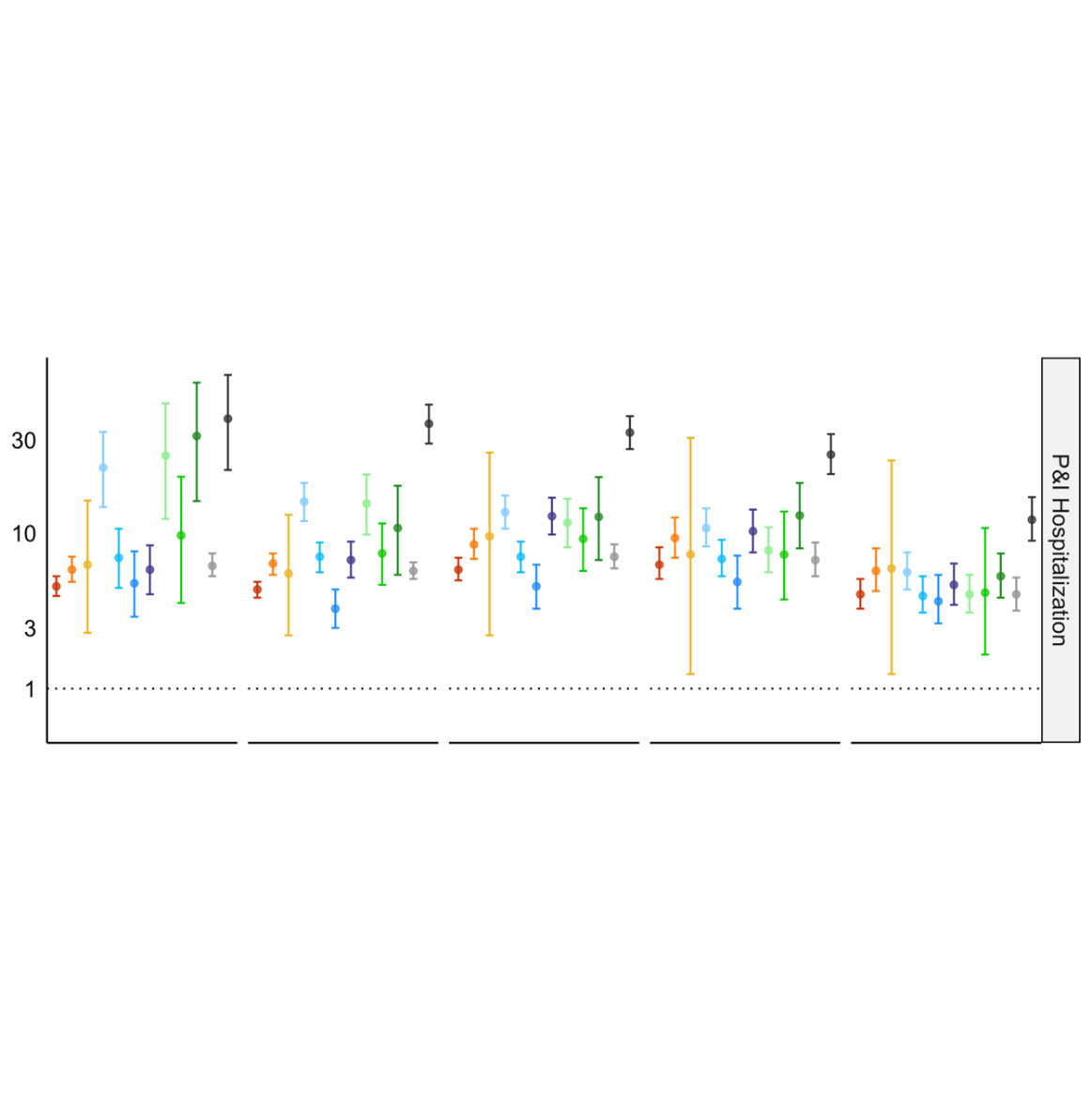

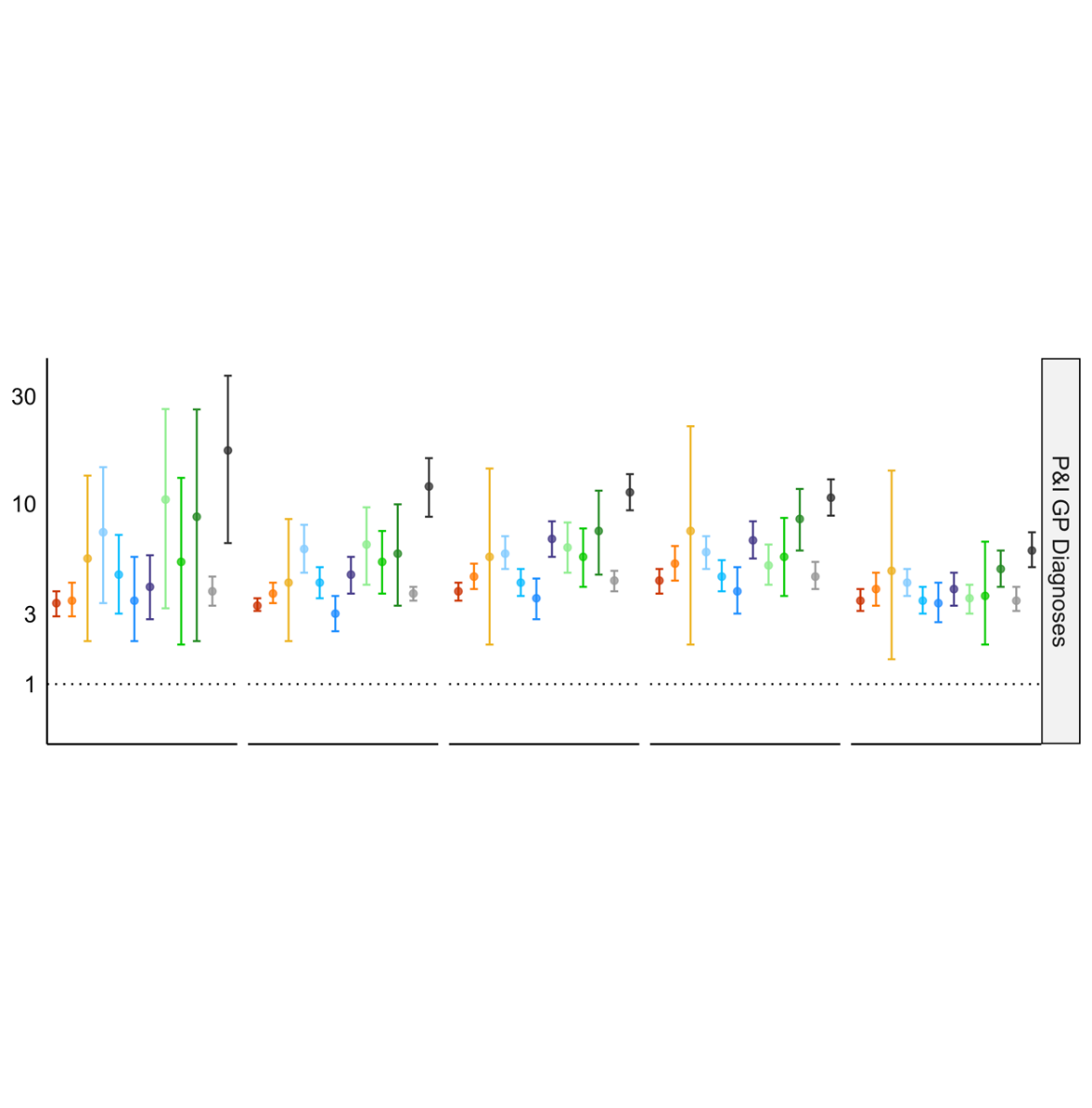

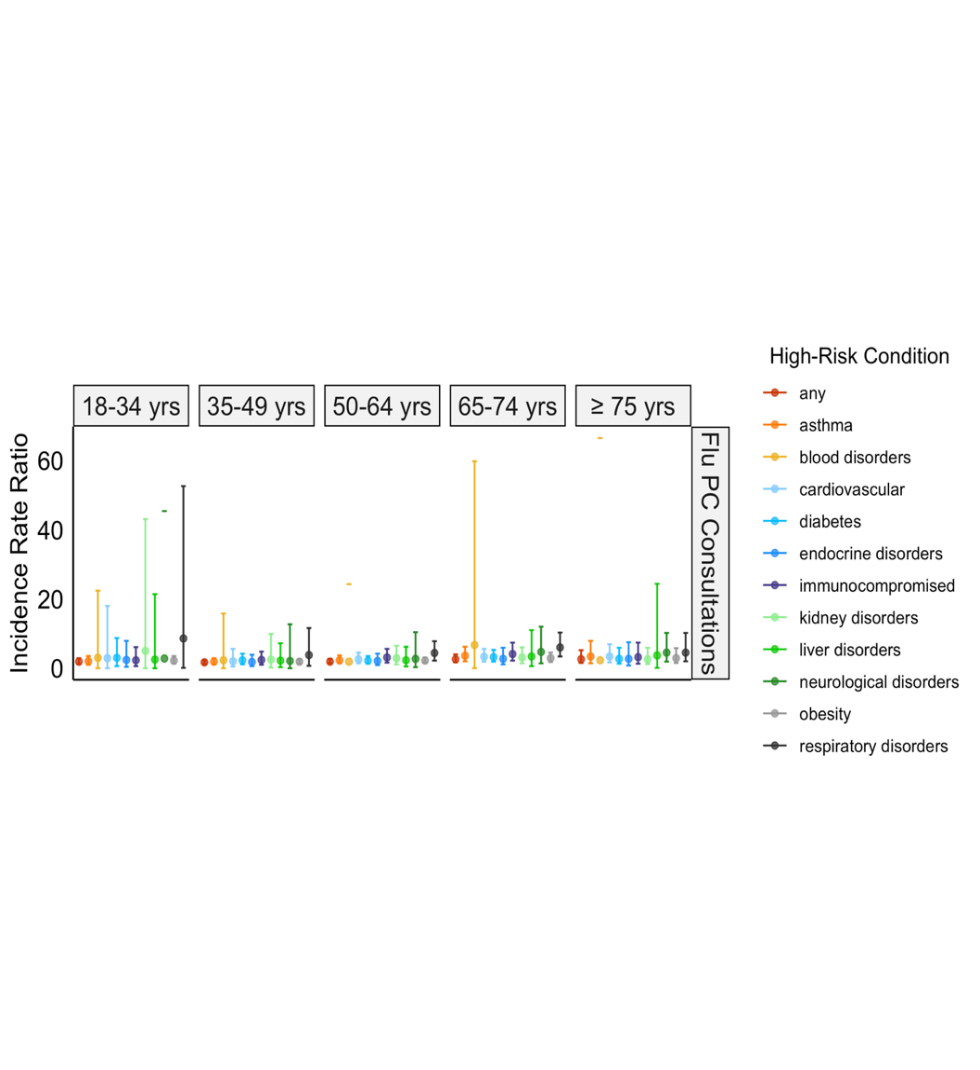

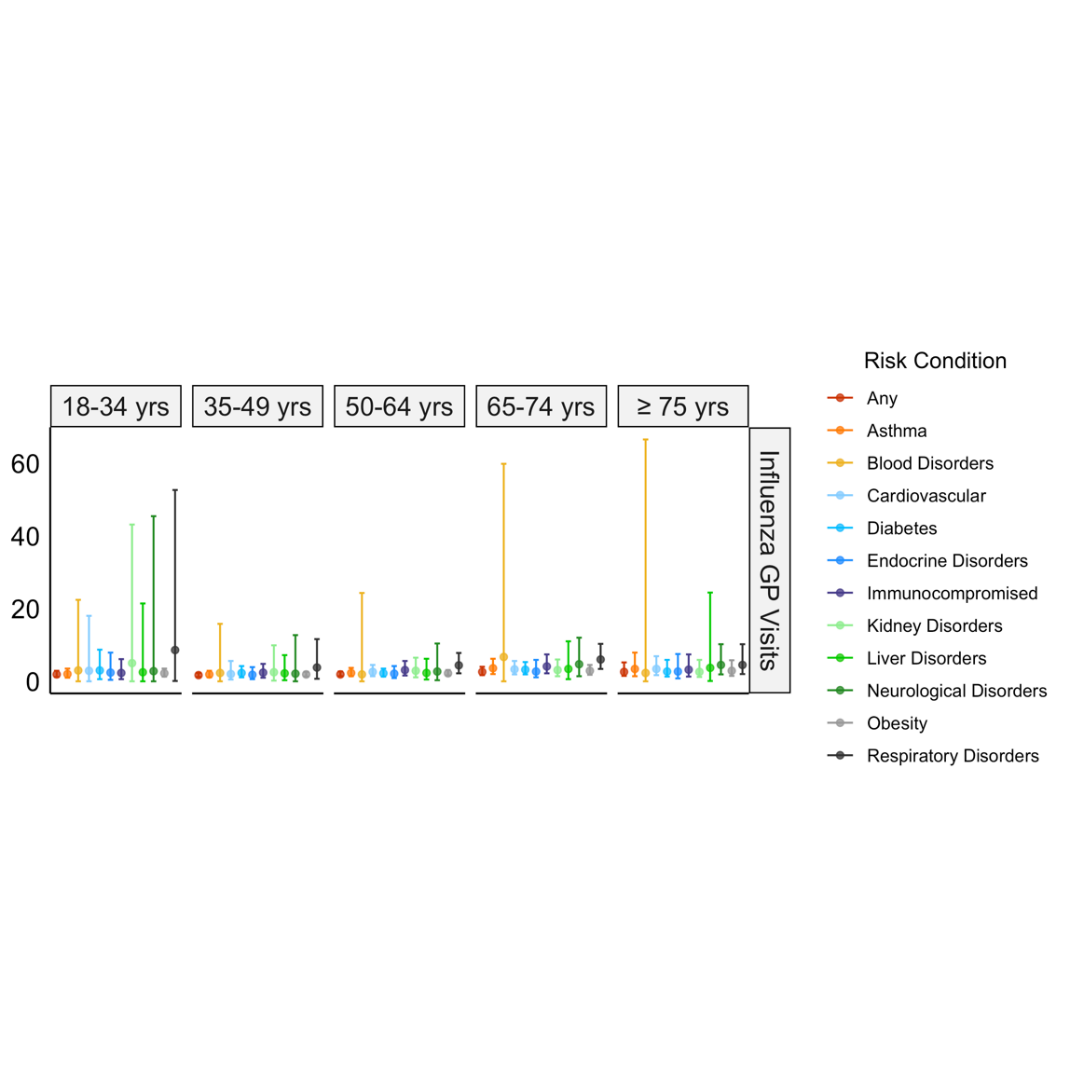


*Abbreviations: yrs = years; any = any risk condition.*
